# Restorative effects and perception of nature-themed artworks in indoor environments: an empirical study based on VR+EEG

**DOI:** 10.3389/fpsyg.2025.1571176

**Published:** 2025-07-09

**Authors:** Jiayin Chen, Huiqiu Zhu, Yue Cheng, Henan Yin, Minzhe Yi, Danni Shen, Zuyao Zhang, Jue Wu

**Affiliations:** ^1^School of Art and Design, Zhejiang Sci-Tech University, Hangzhou, China; ^2^School of Ceramic Art, Jiangxi Arts and Ceramics Technology Institute, Jingdezhen, China; ^3^College of Architecture and Urban Planning, Tongji University, Shanghai, China

**Keywords:** indoor environment, restorative effects, VR, EEG, natural analogues, nature-themed artwork

## Abstract

**Background:**

While numerous studies have examined the restorative effects of natural elements in indoor environments, limited attention has been given to the role of natural analogues. This study investigates the restorative effects of nature-themed artwork, a common natural analogue in biophilic design.

**Materials and methods:**

Virtual reality (VR) was employed to simulate five work environments: rooms featuring nature-themed artwork, a blank foreground, an architectural window view, a natural window view, and a green plant wall, respectively. Measuring content included electroencephalography (EEG), blood pressure, heart rate, and restorative evaluations to compare the effects of these environments on physiological and psychological responses across conditions.

**Results:**

Nature-themed artwork significantly reduced systolic blood pressure (*p* < 0.05), increased alpha wave activity [frontal, central, and occipital regions (*p* < 0.05), and elicited higher restorative evaluation scores compared to the blank foreground and architectural window view]. Positive correlations were found between α power spectral density (PSD) values and restorative evaluation scores (*p* < 0.05). Additionally, no significant differences were found between the data induced by nature-themed artworks, the natural window view, and the green plant wall.

**Discussion:**

The results demonstrate that nature-themed artwork exerts restorative effects comparable to direct natural elements (natural window views and green plant walls), surpassing both blank walls and architectural views. This underscores its potential as an effective natural analogue. This study provides reference information for designers involved in interior natural decoration, considering physiological, neural, and psychological aspects.

## 1 Introduction

Modern work and learning practices often require individuals to spend prolonged periods indoors. The quality of indoor environments significantly influences physical and mental well-being. Extended indoor work can elevate stress levels, potentially causing psychological issues and adverse physiological responses. Consequently, creating indoor environments that promote well-being has become a focal point in architectural and interior design.

In the study of indoor environment, the restorative effect of environmental characteristics is one of the important research topics of environmental psychology. The restorative potential of indoor environments is anchored in two foundational theories: Attention Restoration Theory (ART) and Stress Reduction Theory (SRT). The ART theory points out that natural elements usually have a positive impact on people because they typically conform to the four characteristics of a restorative environment (being away, fascination, extent, and compatibility) (Kaplan, 1995). SRT holds that nature can help people recover from the harmful psycho-physiological effects caused by fatigue and stress. The key factor in this recovery is the positive emotional response triggered by unthreatening and aesthetically pleasing nature, which shapes positive mental and physiological states (Ulrich, 1981).

These theories converge under the Biophilia Hypothesis, which asserts humans’ innate affinity for nature (Wilson, 1986). Biophilic design operationalizes this through direct nature in the space, natural analogues, and nature-mimicking spaces (Kellert et al., 2011). Among them, direct nature in the space mainly refers to plants, water, animals, wind, sounds, smells and other natural elements; natural analogues refer to non-biological elements that indirectly evoke a connection with nature, such as paintings and sculptures with natural themes; spaces mimicking natural processes include types such as those that generate natural circadian rhythms through lighting patterns (Browning et al., 2014; Ghaziani et al., 2021). Numerous studies have demonstrated the restorative effects and positive impacts of incorporating biophilic elements in indoor environments (Yeom et al., 2021; Diette et al., 2003).

As natural analogues, nature-themed artworks, due to their symbolic representation of nature, may offer similar physiological and psychological benefits. Over 160 years ago, Florence Nightingale emphasized the importance of beauty for patients, asserting that it influences both the psyche and physical well-being (Nightingale and Nursing, 1969). In daily life, beauty is often associated with artworks. Many researchers have pointed out that experiencing or viewing artworks can positively impact public health and well-being (Davies et al., 2012; Davies et al., 2015; Cavanagh et al., 2020). These benefits include increased happiness and life satisfaction, improved mental health, and reductions in adverse cardiovascular reactions (Cuypers et al., 2012; Davies et al., 2015). Ulrich and Gilpin (1999) pointed out that artworks in indoor medical environments can improve mood and reduce stress, thereby alleviating the adverse effects of negative thoughts that may hinder recovery. Currently, artworks depicting natural themes are frequently used in interior decoration as symbolic representations of natural elements (Staricoff et al., 2003; Gao and Zhang, 2021). Nature-themed artworks are not only common but also more cost-effective compared to most expensive decoration methods. Therefore, its influence on the perception of the indoor environment deserves in-depth exploration.

Previous studies on natural-themed artworks have primarily focused on the emotional and physiological responses they evoke in people, but there is still a lack of research on their restorative effects. Numerous researchers have found that viewing artworks mimicking natural forms can alleviate stress comparable to experiencing natural landscapes (Gillis and Gatersleben, 2015; Kjellgren and Buhrkall, 2010). Lankston et al. (2010) discovered through a case study of hospital interiors that patients displayed favorable emotional responses towards artworks portraying natural landscapes. Felsten conducted a questionnaire survey and found that college students rated indoor environments adorned with large-scale natural murals significantly higher than those without green landscapes (Felsten, 2009). Responses to nature-themed artworks, Heerwagen and Orians (1990) found that compared to the situation without large natural murals on the walls, the stress level of patients in a dental clinic waiting area were significantly reduced when such murals were present, as reflected in heart rate measurements. Apart from the insufficient exploration in the aspect of restoration effects, previous studies also failed to compare the restoration effects of natural-themed artworks with other restoration elements (such as real window views or plants). Therefore, if it is compared with the restorative effects of other common restorative elements, it can help people accurately understand the restorative effects.

Elements such as architectural window view, natural window view, and green plant are common in the indoor environments. Ulrich once explored the differences in the healing effects of patients between architectural window views and natural window views through a study published in Science (Ulrich, 1984). Green plant wall are also a common topic in indoor environment research (Yeom et al., 2021), and have been proven to be beneficial to people’s perception recovery (Ghamari and Amor, 2016). Thus, in addition to the blank foreground, incorporating different experimental conditions such as architectural window view, natural window view, and green plant wall into the study can more clearly demonstrate the extent to which the restorative effect brought by natural artworks is. Therefore, the variables of the indoor environment in this study were set as five different types of office desk backgrounds: (1) blank foreground (Looking forward from the workplace, there is a blank wall); (2) foreground with architectural window view (Looking forward from the workplace, various architectural views can be seen outside the window); (3) foreground with an nature-themed artwork (Looking forward from the workplace, there is a nature-themed artwork in front); (4) foreground with natural window view (Looking forward from the workplace, one can see the natural landscape outside the window); (5) foreground with green plant wall (Looking forward from the workplace, there is a green plant wall in front).

In terms of the measurement of restorative effects, by collecting and analyzing data such as heart rate, blood pressure and electroencephalogram (EEG), physiological and neural responses can be provided as evidence for subjective evaluation. For instance, Park and Mattson’s study revealed that individuals exhibited lower systolic blood pressure and heart rate in indoor environments adorned with plants compared to typical settings, indicating a heightened sense of relaxation (Park and Mattson, 2009a). EEG can illustrate the influence of environmental features on neural responses. For example, Grassini et al. (2022) found that participants watching videos of beautiful natural environments exhibited significantly higher α power spectral density (PSD) compared to those viewing urban and neutral environment videos, confirming the greater sense of relaxation and comfort induced by natural environment stimuli. However, neurologic evidence on the effects of nature-themed artworks on indoor environmental experience is still lacking. Therefore, considering the significance of restoration effects and the wide application of nature-themed artworks, it is not only necessary to explore their restorative effects, but also to take into account various aspects such as physiological responses, neural activities, and psychological experiences.

Among the various construction methods of the research environment, virtual reality (VR) technology can ensure that the research environment has a certain validity and good controllability. Historically, indoor research environments have included simulation environments, VR environments, and real environments. Some researchers conduct simulated environmental experiments in climate labs, focusing on variables such as spatial scale, light, temperature, and humidity. However, the high costs and space requirements of climate labs make them challenging to implement. Creating multiple environments with different conditions in the real environment also requires significant capital and time. In contrast, VR technology offers a cheaper and faster way to set up research environments. With advancements in VR technology and improvements in the realism of three-dimensional modeling, using VR to study environmental experiences has gained increasing popularity. VR technology is now applied in various fields, including gaming, education, tourism, and architecture (Tian et al., 2021). In recent years, many architectural and environmental design researchers have utilized VR technology to explore user experiences (Taehoon et al., 2019; Li et al., 2021). VR is increasingly used for environmental experience research, demonstrating its capability to induce measurable emotional and restorative responses (Lee et al., 2021; Haeyen et al., 2021; Li et al., 2021). Therefore, this study used VR environments to address the challenges of constructing experimental environments. Furthermore, in order to verify the experience of VR environment, this study will test the sense of presence and fidelity in the experimental materials section.

To understand the influence of nature-themed artworks on people from both subjective and objective perspectives, this experiment will examine the influence of such artworks on environmental restorative quality through physiological, neural response measurements, as well as subjective questionnaire surveys. Specifically, this research addresses the following two research questions:

1. Do significant differences exist in the restorative effects between nature-themed artworks and other interior environmental elements?
2. Are there significant differences in participants’ physiological and neural responses to nature-themed artworks versus other indoor environmental conditions?

Understanding the restorative effects of nature-themed artworks through experiments is crucial in the context of emphasizing the creation of healthy buildings and environments. Therefore, this study will create an environment featuring nature-themed artwork, and try to explore the physiological, neurological and psychological responses of individuals within this environment. These responses will be analyzed by comparing them with those from other environmental conditions using *T*-tests.

## 2 Materials and methods

### 2.1 Experimental materials

**Figure 1:**
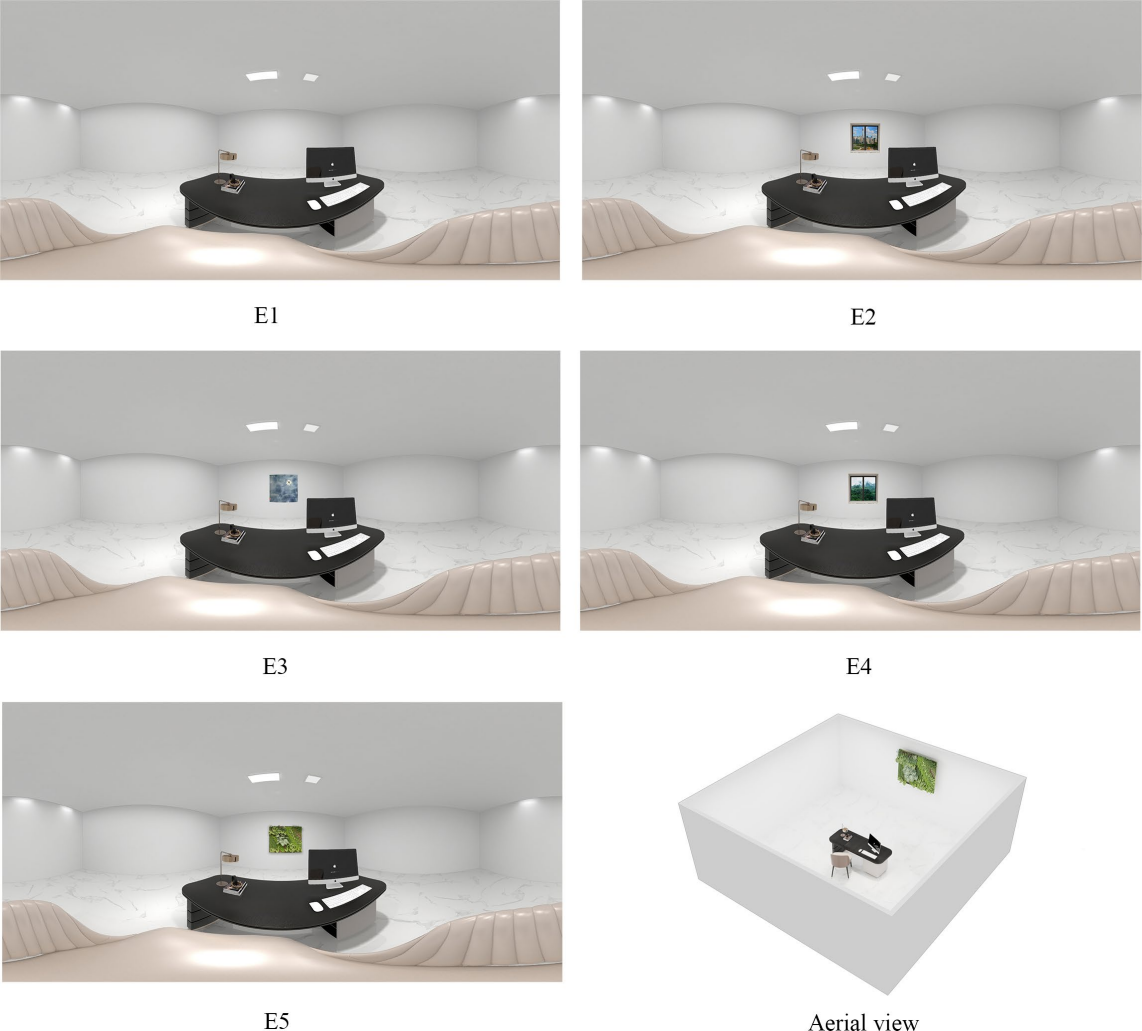
360°panoramic images of five VR environments.

This experiment aims to assess the impact of nature-themed artworks on restorative effects by comparing participants’ responses in different environments. Five virtual reality environments were created: the indoor environment with an empty foreground (E1), the indoor environment with architectural window view (E2), the indoor environment decorated with nature-themed artwork (E3), the indoor environment with natural window view, and the indoor environment with a green plant wall (E5), are shown in Figure 1. The nature-themed artwork is by Chinese artist Bingqin He (with copyright permission), while natural and architectural window views are actual photographs. In previous studies, many scholars incorporated 3D-generated green plant walls into VR environments for environmental perception research (Yeom et al., 2021; Li et al., 2021). Therefore, in order to more realistically represent a plant wall with a certain thickness, we also used 3D software to generate this feature. All the foregrounds such as the architectural window view, nature-themed artwork, natural window view, and green plant wall are all set in the same area and of the same size. Apart from the foreground, the room size, other visual elements, and lighting levels remained consistent across all conditions. The participant’s perspective was set to a tabletop panorama. The inclusion of multiple comparative conditions enables more detailed conclusions regarding the relative restoration potential of nature-themed artworks. To enhance the realism of the experience, the experiment simulated the studio environment using 3D modeling software, VR software, and music player software, addressing both visual and auditory aspects. Each of the five environments was rendered as a 360-degree panoramic image, which was then imported into Unity software for further processing.

Previous studies have shown that the sense of presence (Witmer and Singer, 1998; Gonçalves et al., 2022; Hameed and perkis, 2024) in VR environments and the fidelity (Al-Jundi and Tanbour, 2022) of environmental visual features affect the VR environment experience. In ensure the realism of VR environment experience, this study took E3 as an example and asked participants to assess the environment’s sense of presence and fidelity using a brief questionnaire. Participants rated presence (“Do you feel as though you exist in the VR environment?”) and fidelity (“Are the characteristics realistic?”) on 0–100% scales.

Ten participants were invited to conduct an experience test in the experimental environment. To avoid the potential influence of repeated exposure on the results of the subsequent formal experiment, the ten participants in this test are a separate group, not participants in the subsequent formal experiment. All the participants in this article had no previous VR experience. The test results indicated that the average evaluation score for the E3 environment reached 76.55%, with most participants report that the experience is relatively realistic. Several previous VR studies (Slobounov et al., 2015) have also confirmed the effectiveness of EEG experiments in virtual reality. Based on these results, this study employs VR environments for experiments.

**Figure 2:**
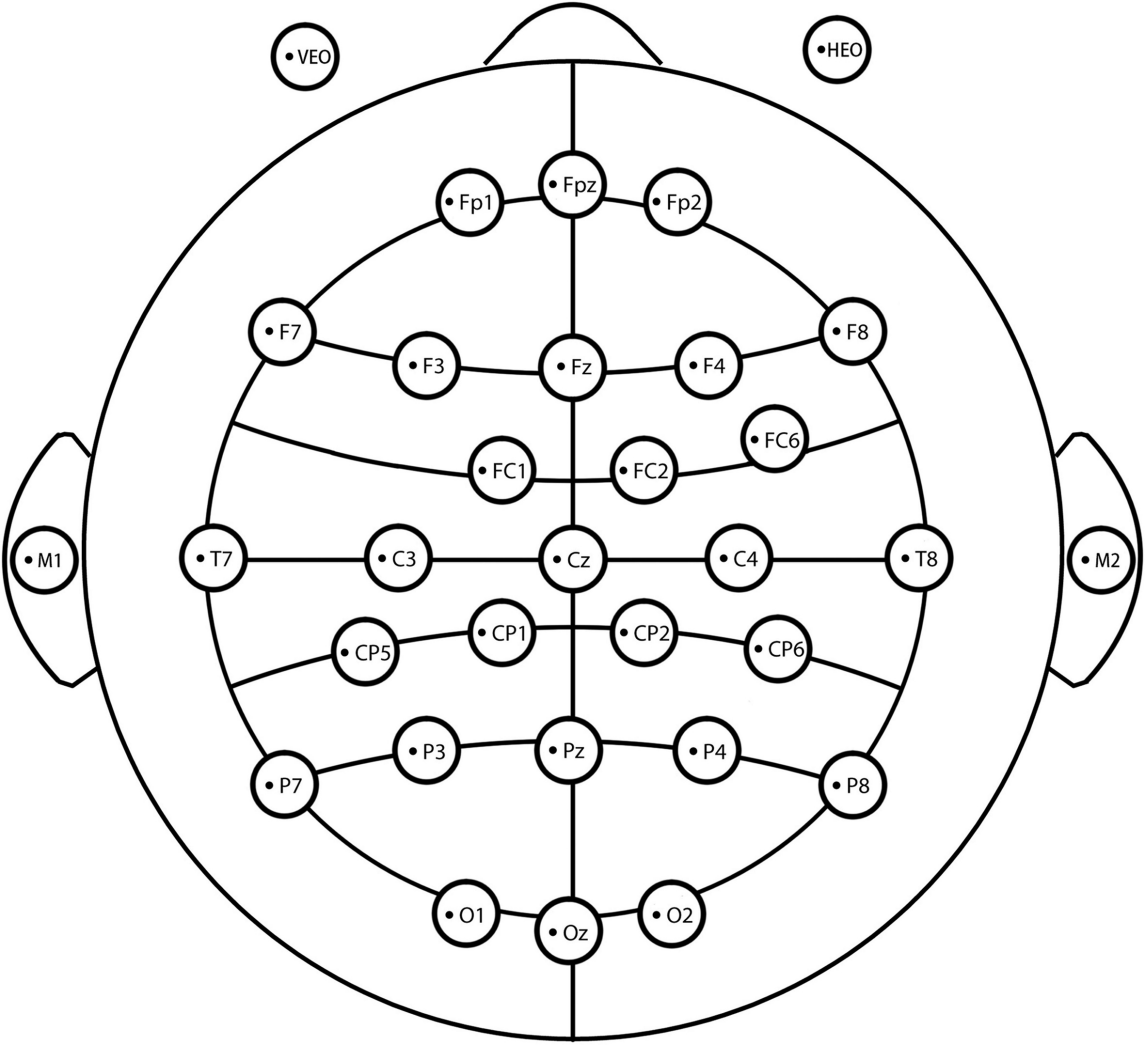
Electrode distribution location map.

Physiological and EEG data were obtained through experimental measurements, while subjective evaluation data were derived from participant scores on the PRS questionnaire. Blood pressure was measured using a Haier blood pressure monitor, and heart rate was recorded with a Meiling finger clip pulse oximeter. EEG signals were continuously recorded using the SMARTING PRO EEG system (32 electrodes), with electrode placement following the extended version of the international 10–20 standard system (Figure 2).

### 2.2 Participants

Based on the estimation results from G*Power software, when α was 0.05 and the power (1-β) was 0.95, a minimal total sample size of 13 was required to detect a medium effect size of 0.4. Thirty university students (15 females) were recruited and offered 70 yuan (approximately $10 USD) for their participation in the experiment. To avoid adverse effects on the results, participants with a history of mental illness, depression, brain disorders, or immune system disorders were excluded. Participants required normal or corrected-to-normal vision (contact lenses only, due to VR headset). All maintained good rest and abstained from stimulants/psychoactive drugs pre-experiment. To induce a baseline state of fatigue/stress, participants completed a challenging 30-min English test (non-native speakers) immediately before the experiment. After completing the test, participants rated their levels of fatigue and stress on a 7-point Likert scale, with the dimensions of “fatigue-relaxed” and “depressed-happy,” where lower scores indicated higher levels of fatigue and depression. In the subsequent experiment, participants’ fatigue and stress levels were measured. Analysis of variance revealed no significant difference (*p* > 0.05) in the fatigue and stress levels of each person before the start of the experiment. Ethical approval was granted by the Ethics Committee of Jingdezhen Third People’s Hospital (LL2023011), in accordance with the Declaration of Helsinki. Informed consent was obtained from all participants.

### 2.3 Assessment content and hypotheses

In this study, the Perceived Restorative Scale (PRS) proposed by Hartig et al. (1997) was used to collect subjective scores of the restorative quality of each environment. Based on ART, Korpela and Hartig expanded the four dimensions (being away, fascination, extent, and compatibility) proposed by Kaplan (1995) and established the PRS scale (Korpela and Hartig, 1996), which have been widely used in restorative environment research. Hartig et al. (1997) revised the scale, using a 7-point Likert scale for measurement. A higher total score on the restoration evaluation indicates a stronger restoration effect of the environment (Su and Xin, 2010). Previous study has shown that decorative paintings with natural themes can enhance the artistic atmosphere of the environment and make people feel relaxed (Gong, 2013). Therefore, we present hypothesis 1: participants will have higher restorative evaluation scores in the environment containing nature-themed artwork compared to the empty foreground environment.

Previous studies on the restorative quality of environment have shown that such environments can promote parasympathetic nerve activity and reduce stress-induced increases in heart rate (Ulrich et al., 1991). Therefore, heart rate variability was included in the physiological measurements. Blood pressure is another key component in the assessment of physiological responses. Diastolic blood pressure (DBP) and systolic blood pressure (SBP) are important parameters of blood pressure, usually measured in millimeters of mercury (mmHg), and serve as critical indicators of cardiovascular system (Taylor et al., 2011). Research has shown that restorative environments can mitigate the increase in blood pressure following stress (Kjellgren and Buhrkall, 2010; Park and Mattson, 2009b). Therefore, examining the systolic and diastolic blood pressure of participants in different environments may provide insight into the effects of the environment in terms of blood pressure response. Thompson (2023) found that nature-themed artworks can alleviate stress and fatigue while promoting physiological recovery. Thus, we proposed Hypothesis 2: compared with the environment with empty foreground, participants’ heart rate and blood pressure are lower in the environment containing nature-themed artwork.

The frequency of the α rhythm ranges from 8 to 13 Hz. In terms of human state regulation, the α rhythm is often associated with relaxed, calm states and is therefore considered an index of relaxation, meditation, concentration, or entering a “flow” state. During deep relaxation exercises, people typically exhibit higher levels of α wave activity. Additionally, some studies have found that α wave activity is related to attention, cognitive ability, and emotional regulation (Sadaghiani et al., 2010). Generally, α waves increase in states of relaxation, meditation, or concentration and decrease in states of anxiety, tension, or fatigue (Sanei and Chambers, 2013). Frontal α waves are generally elevated in people who have a good experience of the environment (Grassini et al., 2022; Chang and Chen, 2005; Chiang et al., 2017). The PSD refers to the signal power at each frequency, that is, the average power of the signal over a bandwidth of 1 Hz. According to Dixit’s (2021) research, nature-themed artworks can evoke positive memories and elicit sustained attention and interest. Therefore, we proposed Hypothesis 3: compared with the empty foreground environment, participants in the environment containing nature-themed artwork will exhibit higher frontal α PSD.

### 2.4 Experimental procedures

**Figure 3:**
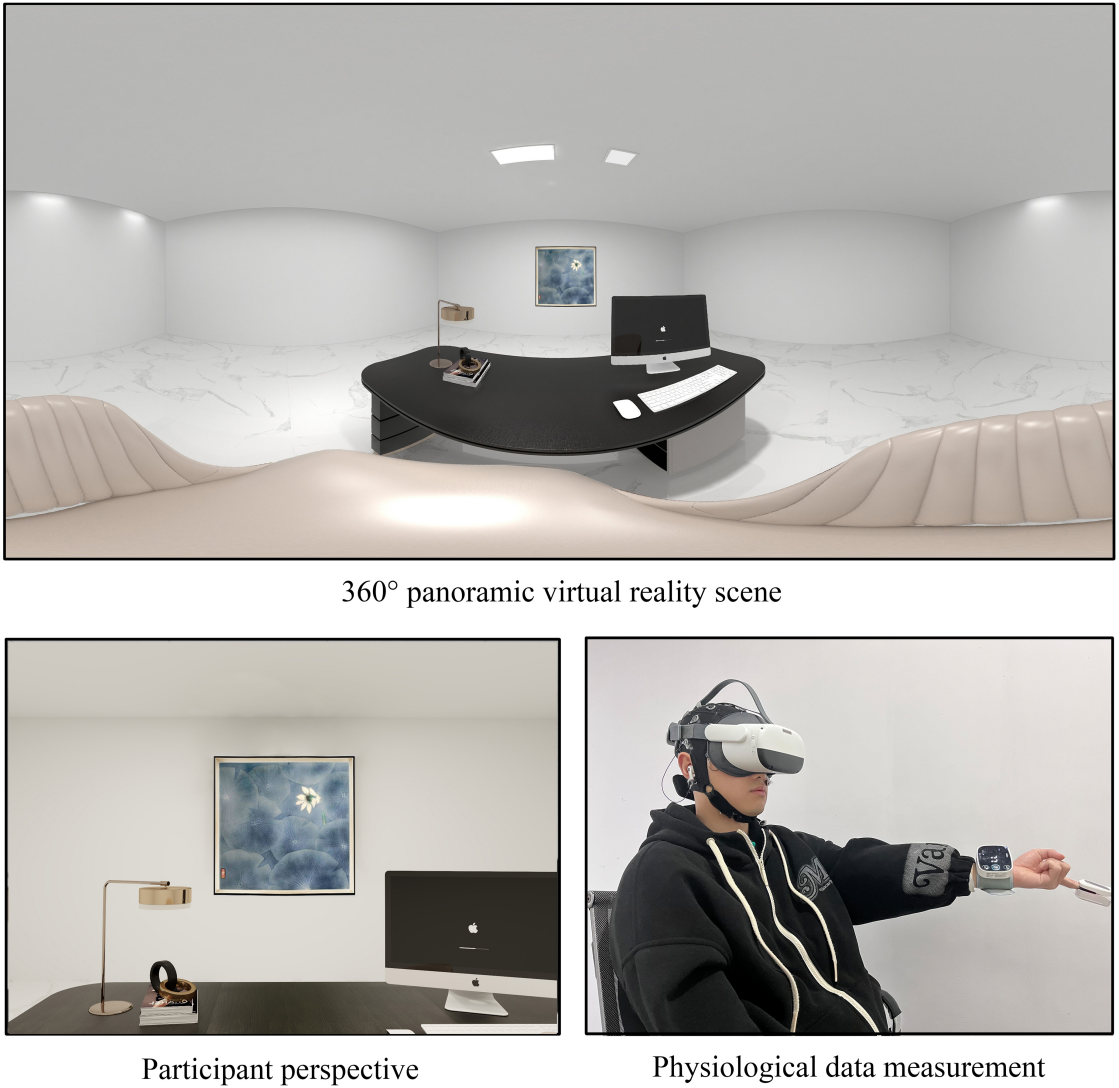
Participants and bystander perspectives.

**Figure 4:**
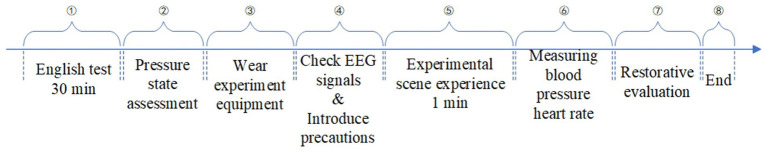
Experimental procedure.

This research mainly studies the effect of nature-themed artwork on the restorative quality of the indoor environments, necessitating the control of other environmental variables. An air quality monitor was used to monitor the experimental environment according to Indoor Air Quality (IAQ) (China State Administration for Market Regulation and Standardization Administration, 2022). During the experiment, the indoor carbon dioxide concentration was maintained below 1,000 ppm, total volatile organic compounds (TVOC) below 500 micrograms per cubic meter, PM2.5 below 12 micrograms per cubic meter, and PM10 below 50 micrograms per cubic meter. According to “Green Building Evaluation Standards” (GB/T 50378–2014) (China State Administration for Market Regulation and Standardization Administration, 2014) and the study of human settlement thermal environment (Frontczak and Wargocki, 2011), the laboratory temperature was set at 23 degrees Celsius, with indoor relative humidity controlled at approximately 50%, and proper ventilation was maintained. For auditory control, noise-canceling headphones were used to block external noise while playing white noise in the office and experimental guidance speech. The procedure for all scenarios was as follows:

(1) To understand the restorative effect on individuals under stress and fatigue, each participant spent approximately 30 min completing a series of English tests before the experiment. (2) A stress state assessment was performed after each participant completing the English test. (3) Participants are fitted with EEG caps, and conductive paste was applied to their scalps. Once the EEG software was activated and the impedance between the electrodes and the scalp was controlled below 5,000 *Ω*, the participant was fitted with VR glass connected to the computer. Researchers examined EEG signals to ensure that participants produced normal eye electricity during blinking and saccades and that there was no baseline drift. (4) Explain the precautions of the experiment to the participants and wear noise-cancelling headphones for them. (5) The formal experiment began. The scene was imported into Unity, and the EEG recording function was activated. Pre-recorded white noise and short introductory remarks were played through the noise-canceling headphones to create a more immersive experience, lasting 1 min. (6) Participants’ blood pressure and heart rate were measured and recorded (Figure 3). (7) A PRS questionnaire was introduced, and participants were asked to evaluate the environment, with their scores recorded. Following these steps, the experiment for each individual scenario was completed. The procedure for all scenario was consistent, with each participant experiencing all five environments. To minimize fatigue effects, each participant completed the experiments for each scenario on separate days. To control for sequential effects, the orders of experimental conditions for each participant was randomized using a Latin square design, that is, each scene was evenly distributed among the experimental sequences. The experimental process for each scenario is shown in Figure 4.

### 2.5 Data recording and processing

#### 2.5.1 Restorative evaluation and physiological data recording

This experiment aims to explore the perceptual restorative differences between indoor environments decorated with nature-themed artwork (E3) and other characteristics. To compare these differences more intuitively, the data generated by participants in the E3 environment were used as the standard group, and the data from the other four environments were subjected to paired sample *T*-tests.

Data from 23 participants (11 M/12F; 7 excluded due to incompleteness) were analyzed using SPSS 19.0. In the restorative quality evaluation analysis, Cronbach’s alpha coefficient was used to test the internal consistency of the questionnaire. The PRS demonstrated good internal consistency (Cronbach’s α = 0.71–0.83 per dimension) and validity (KMO = 0.82, Bartlett’s *p* < 0.001). After calculating the total score for each participant, the data from the E3 environment were used as the control group and paired sample *T*-tests were conducted for the remaining four environments.

#### 2.5.2 EEG data recording and processing

The EEG signals collected in this experiment were processed using power spectral density analysis. The power spectrum density analysis is used to convert the EEG signal from the time domain to the frequency domain to help understand the relationship between energy and signal frequency more directly. This method is commonly used to analyze changes in brain wave rhythm generated by participants.

The data processing in this experiment involved several steps, detailed as follows: (1) Split the data: divide the one-minute time segment according to the time mark in the data after each participant enters different scenes. (2) Remove useless electrodes: Remove useless electrodes such as HEOG and VEOG. (3) Filtering: Band-pass filtering was used to remove baseline drift and power frequency interference, set to frequencies above 1 Hz and below 30 Hz. (4) Re-reference: Use the average value of M1 and M2 reference electrodes as the reference value. (5) Independent component analysis (ICA): Manually remove artifacts such as ophthalmoelectric and myoelectric after running ICA. (6) Fast Fourier transform: Convert the time domain signal into the frequency domain signal. Using the calculation method proposed by Welch (1967), the baseline was set to −2 to 0 s, with a step size of 2 s and an overlap rate was 50% (Hamming window, 1,024 data points). The EEG signals for each time period are Fourier transformed to obtain amplitude and phase information for each frequency. (7) Power spectral density extraction: After extract the average α PSD value generated by participants in different environments and from different electrodes, using the data from the E3 environment as the control group to perform paired sample *T*-tests for the remaining 4 environments. (8) Graphic display: Generate power spectrum maps and topography maps for different environments.

## 3 Results

**Figure 5:**
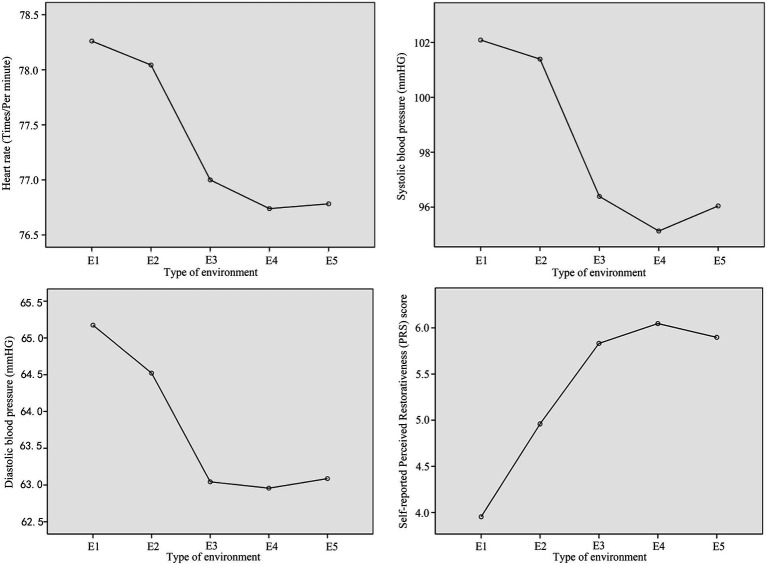
The average values of heart rate, systolic blood pressure, diastolic blood pressure and perceived recovery evaluation triggered by different environmental conditions.

This section presents the paired-sample *T*-test results comparing participants’ subjective evaluations, physiological and neural responses between E3 and the other environments: E1, E2, E4, and E5. Figure 5 shows the heart rate, blood pressure and subjective evaluation results triggered under different experimental conditions (see Table 1).

**Table 1:** Realistic assessment of virtual reality experimental environment.

Factor	Questions	Evaluation range	Score (%)
Presence	Do you feel like you exist in a VR environment?	No–Yes	0–100
Fidelity	Are the characteristics of the environment real?	It’s not real–It’s real	0–100

### 3.1 Physiological measurement results of different indoor environments

#### 3.1.1 Heart rate

Paired sample *T*-test analyses indicated no significant differences in heart rate values between E3 and E1 (*T* = −1.421, *p* = 0.169), E3 and E2 (*T* = −0.897, *p* = 0.38), E3 and E4 (*T* = 0.265, *p* = 0.793), or E3 and E5 (*T* = 0.205, *p* = 0.84). The ranking of mean heart rate values across different environments is as follows: E4 (76.739 bpm) < E5 (76.783 bpm) < E3 (77 bpm) < E2 (78.043 bpm) < E1 (78.261 bpm). Table 2 presents the results of the *T*-test.

**Table 2:** Paired sample *T*-test results between participants’ heart rate values generated in E3 and other environments (times per minute).

Environments	Pairs 1		Pairs 2		*T*	*p*	Cohen’s d
	Mean	SD	Mean	SD			
E3 vs. E1	77	1.925	78.261	1.95	−1.421	0.169	0.296
E3 vs. E2	77	1.925	78.043	1.892	−0.897	0.38	0.187
E3 vs. E4	77	1.925	76.739	1.909	0.265	0.793	0.055
E3 vs. E5	77	1.925	76.783	1.867	0.205	0.84	0.043

#### 3.1.2 Blood pressure

##### 3.1.2.1 Systolic blood pressure

As shown in Table 3, the paired sample *T*-test results for systolic blood pressure indicate significant differences between E3 and E1 (*T* = −3.301, *p* = 0.003), and between E3 and E2 (*T* = −2.912, *p* = 0.008). There are no significant differences between E3 and E4 (*T* = 1.034, *p* = 0.312), or between E3 and E5 (T = 0.357, *p* = 0.724). The ranking of mean systolic blood pressure values of different environments is as follows: E4 (95.13 mmHg) < E5 (96.043 mmHg) < E3 (96.391 mmHg) < E2 (101.391 mmHg) < E1 (102.087 mmHg). Table 3 presents the results of the *T*-test.

**Table 3:** Paired sample *T*-test results for participants’ systolic blood pressure values in E3 and other environments (mmHG).

Environments	Pairs 1		Pairs 2		*T*	*p*	Cohen’s d
	Mean	SD	Mean	SD			
E3 vs. E1	96.391	2.175	102.087	2.333	−3.301	0.003^*^	0.688
E3 vs. E2	96.391	2.175	101.391	2.022	−2.912	0.008^*^	0.607
E3 vs. E4	96.391	2.175	95.13	2.043	1.034	0.312	0.216
E3 vs. E5	96.391	2.175	96.043	1.898	0.357	0.724	0.075

*means *p* <0.05.

##### 3.1.2.2 Diastolic blood pressure

For diastolic pressure, paired sample *T*-tests indicated no significant differences between E3 and E1 (*T* = −2.039, *p* = 0.054), E3 and E2 (*T* = −1.16, *p* = 0.258), E3 and E4 (*T* = 0.055, *p* = 0.957), and E3 and E5 (*T* = −0.037, *p* = 0.971). The ranking of mean diastolic blood pressure values of different environments is as follows: E4 (62.957 mmHg) < E3 (63.043 mmHg) < E5 (63.087 mmHg) < E2 (64.522 mmHg) < E1 (65.174 mmHg). Table 4 presents the results of the *T*-test.

**Table 4:** Paired sample *T*-test results for participants’ diastolic blood pressure values in E3 and other environments (mmHG).

Environments	Pairs 1(mmHG)		Pairs 2(mmHG)		*T*	*p*	Cohen’s d
	Mean	SD	Mean	SD			
E3 vs. E1	63.043	1.585	65.174	1.512	−2.039	0.054	0.425
E3 vs. E2	63.043	1.585	64.522	1.506	−1.16	0.258	0.242
E3 vs. E4	63.043	1.585	62.957	1.572	0.055	0.957	0.011
E3 vs. E5	63.043	1.585	63.087	1.712	−0.037	0.971	0.008

### 3.2 Results of restorative quality evaluations

The paired sample *T*-test results for restorative quality evaluations indicated significant differences between E3 and E1 (*T* = 6.153, *p* < 0.001), as well as between E3 and E2 (*T* = 3.153, *p* = 0.005). Conversely, no significant differences were found between E3 and E4 (*T* = −1.373, *p* = 0.183), or between E3 and E5 (*T* = −0.357, *p* = 0.725). The ranking of mean subjective evaluation scores of different environments is as follows: E4 (6.046) > E5 (5.898) > E3 (5.832) > E2 (4.96) > E1 (3.953). Table 5 presents the results of the *T*-test.

**Table 5:** Paired sample *T*-test results of participants’ restorative quality assessment scores for E3 and other environments.

Environments	Pairs 1		Pairs 2		*T*	*p*	Cohen’s d
	Mean	SD	Mean	SD			
E3 vs. E1	5.832	1.09	3.953	0.823	6.153	< 0.001^*^	1.431
E3 vs. E2	5.832	1.09	4.96	1.041	3.153	0.005	0.57
E3 vs. E4	5.832	1.09	6.046	0.991	−1.373	0.183	0.448
E3 vs. E5	5.832	1.09	5.898	0.794	−0.357	0.725	0.094

*means *p* <0.05.

### 3.3 α PSD values

**Figure 6:**
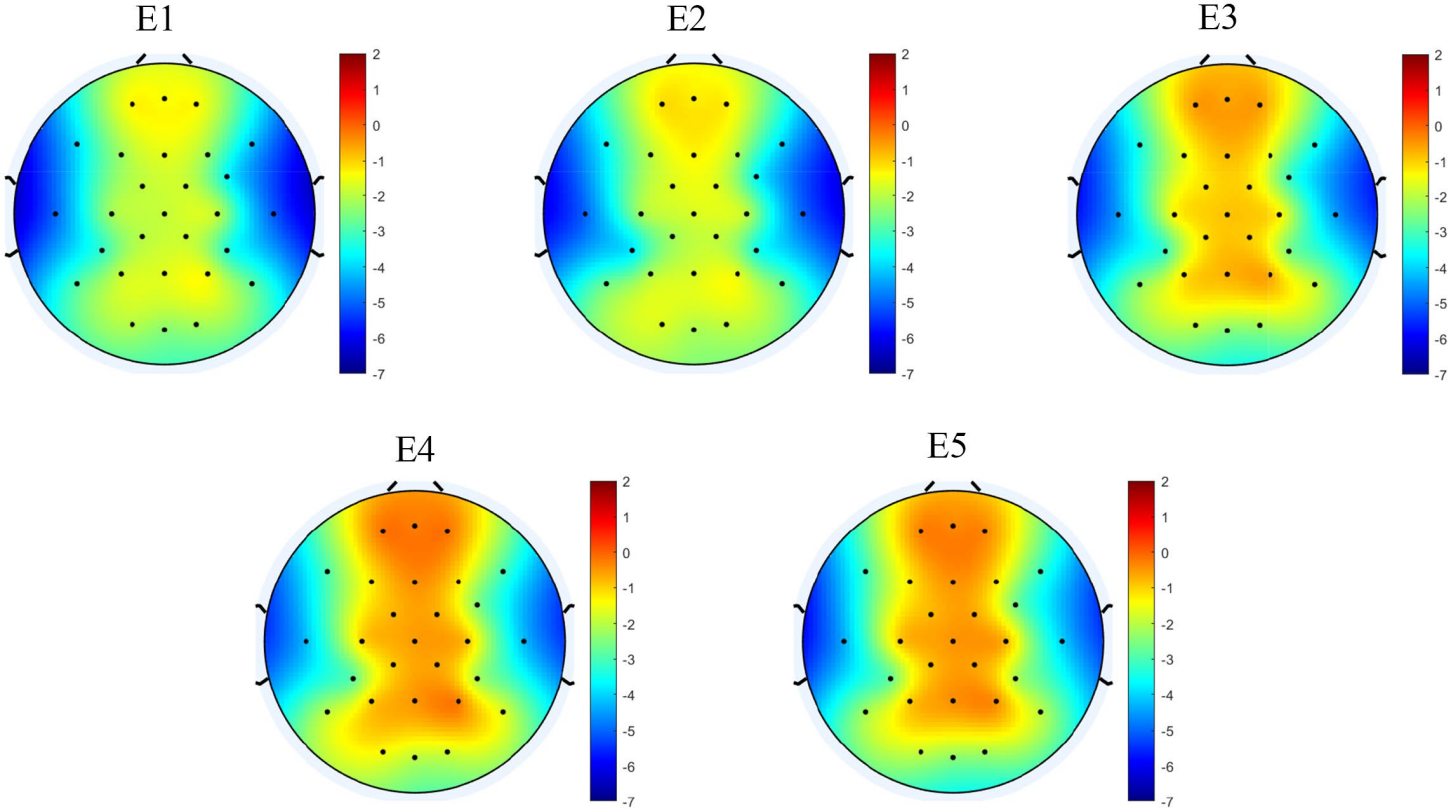
Brain topographic map in the alpha wave frequency band.

**Figure 7:**
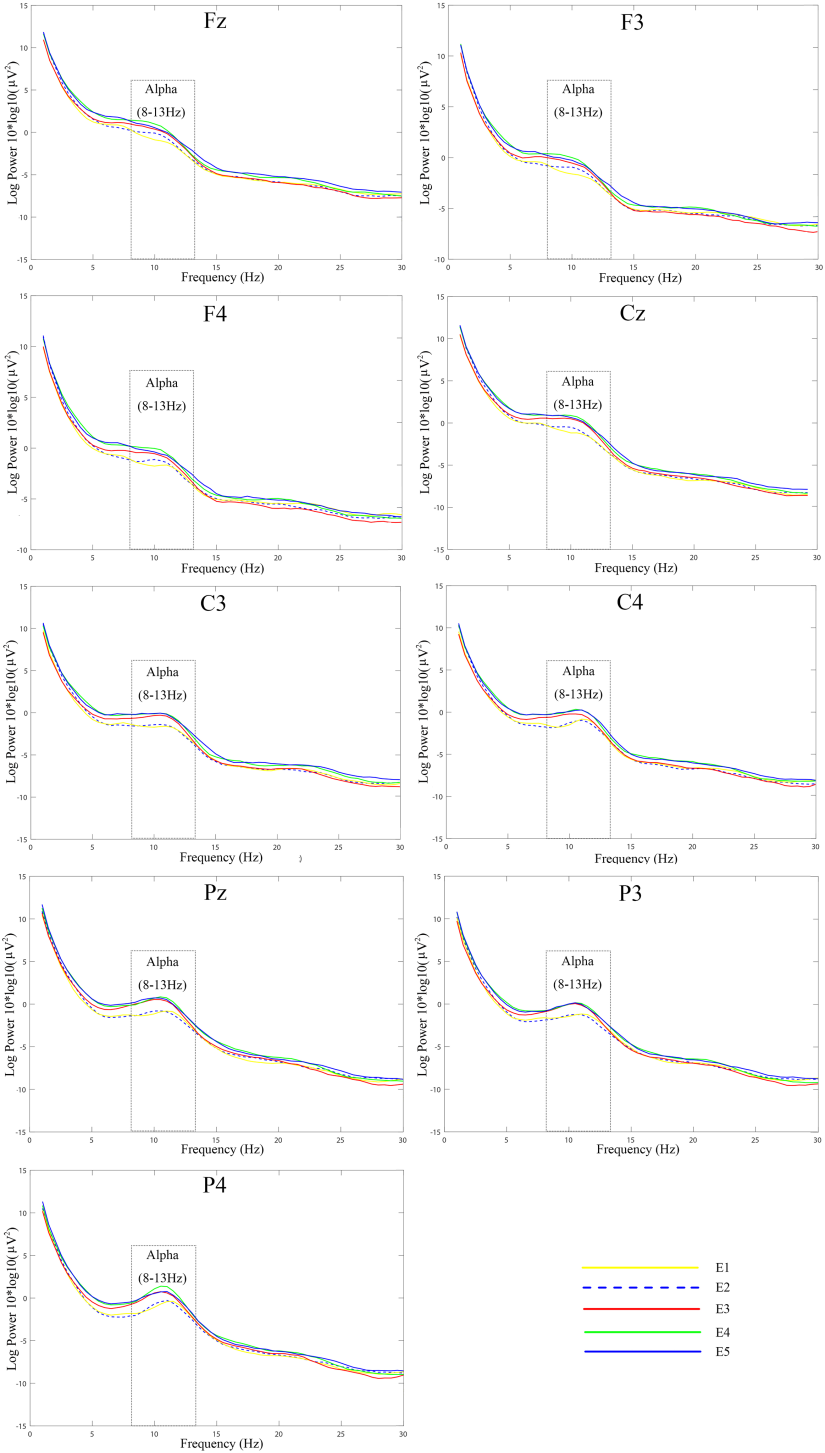
Alpha wave power spectra of different regions.

**Figure 8:**
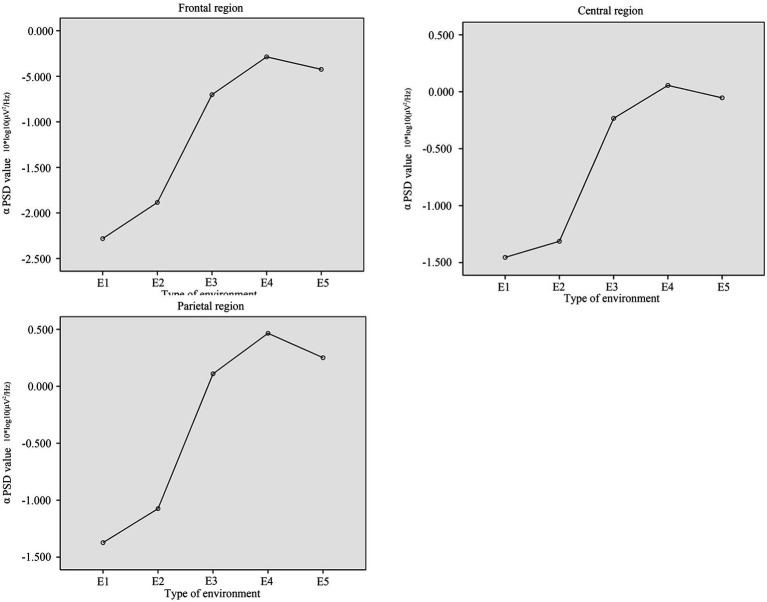
The average values of α PSD in the frontal, central, and occipital.

Topographic and power spectrum maps revealed increased α wave activity across the frontal, central, and parietal regions during resting and relaxation in different environments. No significant differences were observed in α PSD values recorded from the occipital region electrodes under the five experimental conditions. Therefore, this section focuses on the differences in α PSD values across the frontal, central, and parietal regions among participants in these conditions. Additionally, the correlation between α PSD values and subjective restorative evaluation scores are presented. The topography map for the α band is shown in Figures 6, 7 displays the power spectrum (1–30 Hz) of the frontal, central, and parietal regions. Figure 8 illustrates these values in different regions, with the vertical axis representing α PSD values and the horizontal axis denoting the environment types.

#### 3.3.1 Frontal region

The paired sample *T*-test for α PSD in the frontal region showed significant differences between E3 and E1 (*T* = 2.151, *p* = 0.043), and between E3 and E2 (*T* = 2.142, *p* = 0.044). No significant differences were found between E3 and E4 (*T* = −0.838, *p* = 0.411), or between E3 and E5 (*T* = −0.499, *p* = 0.623). The average α PSD values for different environments is as follows: E4 (−0.287) > E5 (−0.424) > E3 (−0.701) > E2 (−1.885) > E1 (−2.281). Table 6 presents the results of the *T*-test.

**Table 6:** Paired sample *T*-test results between participants’ α PSD in frontal regions (Fz, F3, F4) triggered by E3 and other environments [10*log10(μV^2^/Hz)].

Environments	Pairs 1		Pairs 2		*T*	*p*	Cohen’s d
	Mean	SD	Mean	SD			
E3 vs. E1	−0.701	0.648	−2.281	0.644	2.151	0.043^*^	0.448
E3 vs. E2	−0.701	0.648	−1.885	0.505	2.142	0.044^*^	0.447
E3 vs. E4	−0.701	0.648	−0.287	0.814	−0.838	0.411	0.175
E3 vs. E5	−0.701	0.648	−0.424	0.859	−0.499	0.623	0.104

*means *p* <0.05.

#### 3.3.2 Central region

In the central region, the paired sample *T*-test showed significant differences in α PSD values between E3 and E1 (*T* = 3.598, *p* = 0.002), and between E3 and E2 (*T* = 2.522, *p* = 0.018). However, no significant differences were observed between E3 and E4 (*T* = −0.77, *p* = 0.45), or between E3 and E5 (*T* = −0.414, *p* = 0.683). The ranking of average α PSD value across different environments is as follows: E4 (0.056) > E5 (−0.053) > E3 (−0.233) > E2 (−1.313) > E1 (−1.454). Table 7 presents the results of the *T*-test.

**Table 7:** Paired sample *T*-test results between participants’ α PSD values in central regions (Cz, C3, C4) triggered by E3 and other environments [10*log10(μV^2^/Hz)].

Environments	Pairs 1		Pairs 2		*T*	*p*	Cohen’s d
	Mean	SD	Mean	SD			
E3 vs. E1	−0.233	0.415	−1.454	0.515	3.598	0.002^*^	0.75
E3 vs. E2	−0.233	0.415	−1.313	0.406	2.522	0.018^*^	0.532
E3 vs. E4	−0.233	0.415	0.056	0.544	−0.77	0.45	0.161
E3 vs. E5	−0.233	0.415	−0.053	0.57	−0.414	0.683	0.086

*means *p* <0.05.

#### 3.3.3 Parietal region

The paired sample *T*-test for the parietal region revealed significant differences in α PSD values between E3 and E1 (*T* = 2.163, *p* = 0.042), and between E3 and E2 (*T* = 2.686, *p* = 0.013). However, no significant differences were found between E3 and E4 (*T* = −0.77, *p* = 0.449), or between E3 and E5 (*T* = −0.207, *p* = 0.838). The ranking of average α PSD value across different environments is as follows: E4 (0.465) > E5 (0.247) > E3 (0.11) > E2 (−1.074) > E1 (−1.373). Table 8 presents the results of the *T*-test.

**Table 8:** Paired sample *T*-test results between participants’ α PSD values in the parietal region (Pz, P3, P4) triggered by E3 and other environments [10*log10(μV^2^/Hz)].

Environments	Pairs 1		Pairs 2		*T*	*p*	Cohen’s d
	Mean	SD	Mean	SD			
E3 vs. E1	0.11	0.616	−1.373	0.777	2.163	0.042^*^	0.451
E3 vs. E2	0.11	0.616	−1.074	0.578	2.686	0.013^*^	0.56
E3 vs. E4	0.11	0.616	0.465	0.835	−0.77	0.449	0.161
E3 vs. E5	0.11	0.616	0.247	0.739	−0.207	0.838	0.075

*means *p* <0.05.

#### 3.3.4 Overall trends and correlation analysis

Across the frontal, central, and parietal regions, the α PSD values consistently followed the trend: E4 > E5 > E3 > E2 > E1. Despite variations in average values along the vertical axis, this pattern remained consistent across all regions. Pearson correlation analysis revealed significant positive correlations between the α PSD values and the restorative evaluation scores in the frontal (*p* = 0.014, *r* = 0.506), central (*p* = 0.038, *r* = 0.436), and parietal (*p* = 0.022, *r* = 0.475) regions. These results suggest there may be a potential link between subjective restorative evaluations and α wave activity.

## 4 Discussion

Physiological data indicate lower systolic blood pressure at rest in environments with nature-themed artwork versus empty foregrounds. Although no significant differences were observed in heart rate or diastolic blood pressure across the various environments, participants in condition E3 showed marginally lower heart rates and diastolic blood pressure than those in E1 and E2. As stress elevates blood pressure short-term (Nwanaji-Enwerem et al., 2022), and restorative environments promote relaxation and parasympathetic activation, reducing physiological indicators like BP (Yin et al., 2020). The findings of the current study suggest that the presence of nature-themed artwork in indoor environments can contribute to the restoration from physiological stress. Thus, the physiological data largely support Hypothesis 2. In contrast to previous studies (Elsadek et al., 2019; Elsadek and Liu, 2021), the present study provides evidence that environments featuring nature-themed artwork can reduce physiological arousal by promoting perceptual recovery. Furthermore, the inconspicuous differences caused by various environments may be due to the fact that the influence of environmental characteristics has not reached a sufficient degree to cause large differences.

The sequencing of α PSD and subjective restorative evaluations observed in the results follows a consistent pattern: E4 > E5 > E3 > E2 > E1. The positive correlation between α PSD and restorative evaluation scores further substantiates the relationship between these two variables. These findings suggest that α PSD serves as an effective indicator of the restorative evaluation of indoor environments. Moreover, the results revealed no significant differences in restorative evaluations among environments featuring nature-themed artwork, natural window view, and green plant wall. This suggests that nature-themed artwork has comparable restorative effects to natural elements. Furthermore, regardless of whether people were in an environment decorated with nature-themed artwork, natural window view, or green plant wall, α PSD values in the frontal, central, and parietal regions, as well as restoration evaluation scores, were higher than those in environments with an architectural window view. These results not only support Hypotheses 1 and 3, but also contribute new evidence to the understanding of indoor environments, affirming that natural elements in urban environments are more relaxing and exhibit superior restorative effects than built elements (Elsadek and Liu, 2021; Ulrich, 1981). Furthermore, although the experimental materials used in this study differ from those employed in previous research (Grassini et al., 2022; Chen et al., 2016), the results reinforce the notion that α waves increase as individuals become more relaxed.

The findings align with ART and SRT frameworks, where nature-themed artworks function as effective natural analogues by engaging fascination (ART) and eliciting positive emotional responses (SRT). The observed increase in frontal α PSD values (indicative of relaxed attention) and reduced systolic blood pressure suggest that nature-themed art mimics the restorative mechanisms of direct natural elements. Similarly, systolic blood pressure reduction aligns with SRT’s emphasis on parasympathetic activation during exposure to nature-like stimuli. Notably, the lack of significant differences between E3, E4, and E5 underscores the substitutive potential of natural analogues in biophilic design, particularly in settings where direct nature access is constrained. The restorative effects of these biophilic elements may be explained by ART (Berman et al., 2008) and SRT (Ulrich et al., 1991). According to ART, physical and mental states can be effectively restored in environments that possess four key characteristics of restorative perception: being away, fascination, extent, and compatibility (Kaplan, 1995). Such environments help individuals who are constantly consuming attention to relieve stress and recover from attentional fatigue. In addition, according to the SRT, tension and pressure can lead to negative emotions and short-term physiological changes. In environments containing natural elements, attention is naturally drawn to these features, which helps block negative thoughts and elicit positive emotions. When these positive emotions are sufficiently stimulated, they relieve previously negative emotional states and lead to a reduction in physiological arousal. The speed of perceptual recovery is influenced by the restorative strength of the environmental features (Ulrich et al., 1991). Therefore, according to explanations of ART and SRT, participants in environments featuring nature-themed artwork, natural window views, and green plants exhibited higher α PSD values, increased restorative scores, and lower physiological arousal levels. These effect can be attributed to the participants feeling more relaxed after observing and resting in these environments. As stress is alleviated, their physiological states show improved recovery. In contrast to previous studies on nature-themed artwork (Heerwagen and Orians, 1990; Felsten, 2009; Lankston et al., 2010), this study further investigates the impact of natural artwork on indoor environmental experiences from the perspective of neural responses.

## 5 Conclusion

This study advances biophilic design research by demonstrating that nature-themed artwork, as a natural analogue, elicit restorative effects comparable to direct natural elements such as plant and natural window view. Using EEG and physiological metrics, it provides neurobiological evidence supporting ART and SRT, highlighting α wave activity and systolic blood pressure as sensitive indicators of restorative effects. These findings offer practical guidance for designing indoor environments in urban settings where direct nature access is limited, advocating for nature-themed art as a cost-effective and scalable biophilic intervention. Specifically, five distinct VR environments were designed and modeled, and participants’ heart rates, blood pressures, and EEG data were collected through VR+EEG experiments. Paired-sample *T*-tests compared data from the nature-themed artwork environment with other environments, yielding three conclusions:

1. Participants exhibited lower systolic blood pressure, higher α PSD values (in the frontal, central, and occipital regions), and higher restorative scores in environments featuring nature-themed artwork, natural window view, or green plant wall compared to environments with blank foreground or architectural window view. The correlation between α PSD and restorative evaluation scores was statistically significant.
2. Although the systolic blood pressure observed in environments with natural window view and green plant wall were lower than that in the environment with nature-themed artwork, and although α PSD values and restorative evaluation scores were slightly higher that of nature-themed artwork, the differences were not statistically significant. Therefore, it can be suggested that the nature-themed artwork used in this experiment has restorative effects comparable to those of natural window view and green plant wall.
3. From the perspectives of subjective evaluation and physiological response, this study confirms that, as a natural analogue, nature-themed artwork exhibits restorative effects comparable to those of natural elements in biophilic design.

Based on the findings, this study suggests that nature-themed artwork can enhance the restorative quality of indoor environments, particularly in spaces without window or those with only architectural window view.

In an era of increasing work pressure, incorporating appropriate indoor environmental elements is crucial for alleviating stress and fatigue. This research does not evaluate artistic value but explores dimensions such as physiological and neural responses, as well as restorative evaluation. These insights provide a valuable perspective for comparing the restorative effects of different indoor environmental elements, and can provide a basis for the micro-renewal of indoor environment.

## 6 Limitation

Although 23 valid datasets meet the requirements of result validity, our future research will aim to recruit a larger and more diverse sample to obtain more comprehensive data. This study focused exclusively on specific nature-themed artwork. Future investigations will explore the restorative effects of various nature-themed artworks to enhance the generalizability of the findings. Due to the regulations on laboratory usage time, each participant was exposed to different conditions for a limited period of time. Our future research will explore the potential long-term effects of exposure to nature-themed artworks to enhance the ecological validity of the related studies.

## Data Availability

The raw data supporting the conclusions of this article will be made available by the authors, without undue reservation.
